# Flexible wearable sensors - an update in view of touch-sensing

**DOI:** 10.1080/14686996.2020.1862629

**Published:** 2021-03-31

**Authors:** Chi Cuong Vu, Sang Jin Kim, Jooyong Kim

**Affiliations:** Department of Organic Materials and Fibers Engineering, Soongsil University, Seoul, Republic of Korea

**Keywords:** Flexible wearable sensors, touch-sensing, nanomaterials, *e*-skins, *e*-textiles, *e*-healthcare, *e*-control, 10 Engineering and Structural materials, 208 Sensors and actuators, 212 Surface and interfaces

## Abstract

Nowadays, much of user interface is based on touch and the touch sensors have been common for displays, Internet of things (IoT) projects, or robotics. They can be found in lamps, touch screens of smartphones, or other wide arrays of applications as well. However, the conventional touch sensors, fabricated from rigid materials, are bulky, inflexible, hard, and hard-to-wear devices. The current IoT trend has made these touch sensors increasingly important when it added in the skin or clothing to affect different aspects of human life flexibly and comfortably. The paper provides an overview of the recent developments in this field. We discuss exciting advances in materials, fabrications, enhancements, and applications of flexible wearable sensors under view of touch-sensing. Therein, the review describes the theoretical principles of touch sensors, including resistive, capacitive, and piezoelectric types. Following that, the conventional and novel materials, as well as manufacturing technologies of flexible sensors are considered to. Especially, this review highlights the multidisciplinary approaches such as *e*-skins, *e*-textiles, *e*-healthcare, and *e*-control of flexible touch sensors. Finally, we summarize the challenges and opportunities that use is key to widespread development and adoption for future research.

## Graphical abstract

**Figure:**
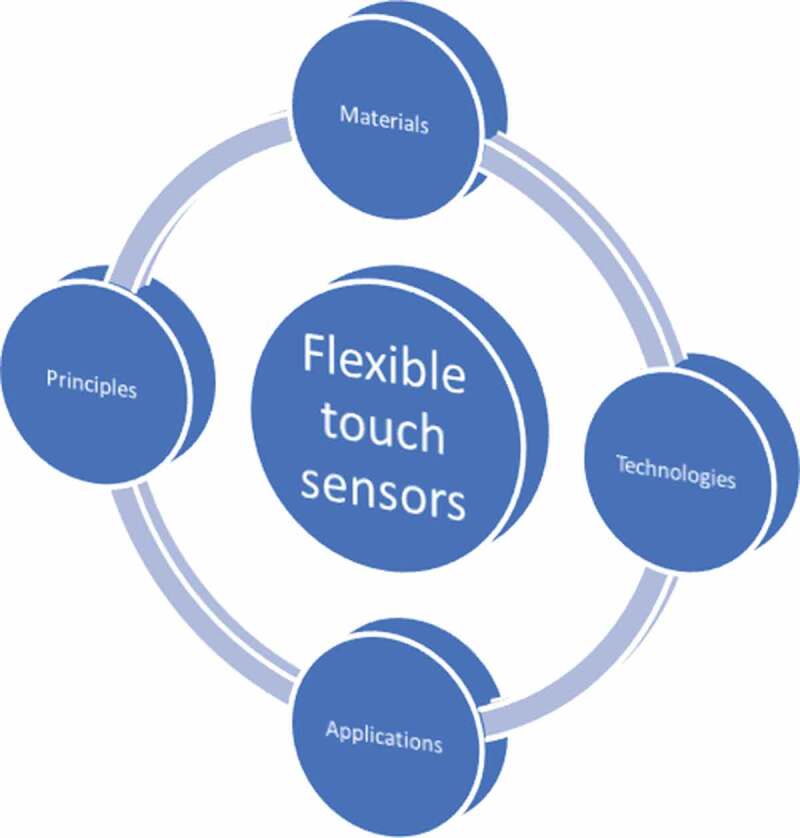

## 1. Introduction

The fourth industrial revolution brings new development steps with many opportunities and challenges, where a lot of data will be collected and analyzed to create new values from life aspects. Following this trend, wearable sensors also become popular. Among them, there are devices capable of monitoring human motions, designing human-machine interfaces, detecting the toxic agents in the environment, or applying in the healthcare/therapeutic [1–3].

Typically fabricated from rigid materials (metals or semiconductors), the traditional sensors are hard, inflexible, and hard-to-wear devices. Meanwhile, flexible sensors show benefits that are superior to traditional rigid devices. These sensors are lightweight, hypoallergenic, and comfortable on the body [4–6].

Flexible touch sensors, as an aspect of flexible wearable sensors, have recently attracted the attention of researchers on the world. There are many studies about the flexible sensors in human motion recognition [7,8], health monitoring of a patient [9,10], or electronic skins [11,12].

**Figure 1.**
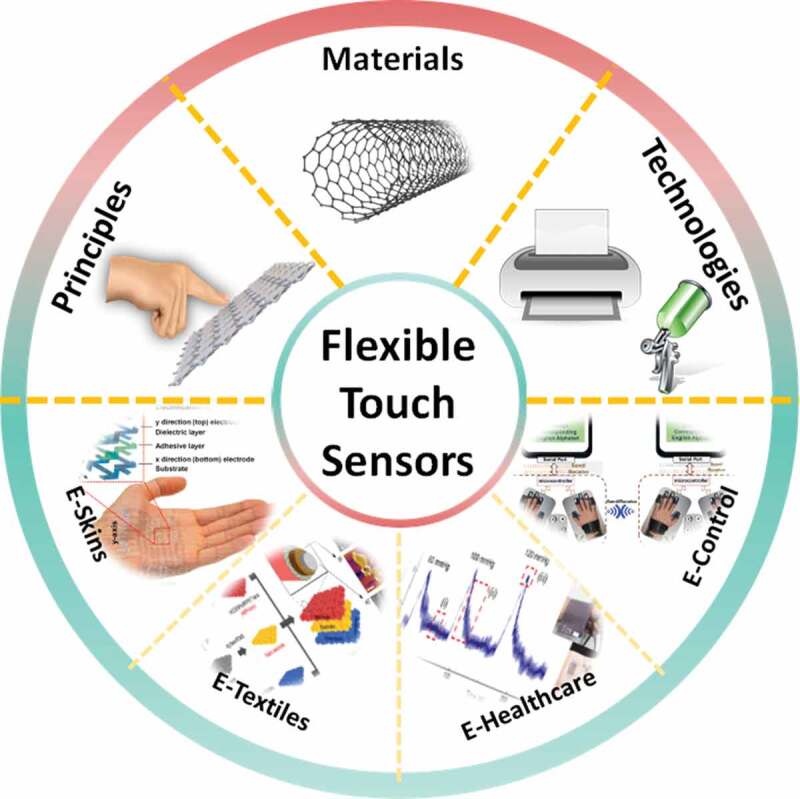
Flexible touch sensors, consisting of Principles, Materials, Technologies, and Applications in *e*-skins. Reproduced with permission from Ref [6]. copyright 2019 Elsevier B.V., *e*-textiles. Adapted with permission from Ref [11]. copyright 2018 Springer Nature, *e*-healthcare. Adapted with permission from Ref [7]. copyright 2017 American Chemical Society, *e*-control. Adapted with permission from Ref [5]. copyright 2020 IEEE

In this short review, we focus only on flexible sensors in view of touch-sensing, emphasizing the sensors incorporated into garments, or directly on the skin for multidisciplinary applications on the latest advancements of recent years, as described in Figure 1. Some basic principles of touch sensors are introduced in Sections 1–2, consisting of capacitive touch, resistive touch, piezoelectric touch, and triboelectric touch. Section 3 reviews the sensing materials, focusing on some types of nanomaterials with many outstanding advantages. Manufacturing technologies are considered in Section 4 with key-methods of current studies. Significant approaches are shown in Section 5. This is also the most important part which updates the research fields and applications of flexible touch sensors into aspects of life. From these advances, we will present a discussion about the next steps in order to bring the sensors from the laboratory to industrial manufacturing (Section 6).

## 2. Working principles of touch sensors

Touch sensing, movement sensing, and pressure sensing are three different types of the sensing-actions. These can be distinguished by the distance between the finger and the sensors, as well as the level of pressure. For normal touch, that distance is 0, and no force applies to the sensors. The level of force will increase with pressure (pressure sensing). The movement sensors can work by a combination of many sensing-points or proximity sensing (current distance will be greater than 0). Herein, we focus on the touch-motions, where the distance is zero and the pressure is relatively small (< 10 kPa). We summarize some different touch sensors according to materials, working principles, electrical/mechanical performances, and thickness (Table 1).

**Table 1.** Summary the electrical and mechanical performance of the different sensors and sensing technologies, where PDMS is polydimethylsiloxane, CNTs are carbon nanotubes, SWNTs are single-walled carbon nanotubes, ITO is indium tin oxide, CNFs are carbon nanofibers, PPy is polypyrrole, BCZT is (Ba_0.85_Ca_0.15_)(Ti_0.90_Zr_0.10_)O_3_, AgNWs are silver nanowires

Ref.	Material	Principle	Sensitivity	Response time	Working range	Thickness
[7]	PDMS, SWNTs, Si, PET	Resistive	1 kPa^−1^	20 ms	0.1–100 kPa	~ 900 µm
[13]	Single-layer ion gel, Copper electrodes	Capacitive	2.266 kPa^−1^	< 1s	1–25 kPa	> 200 µm
[14]	Au/PET, PDMS	Capacitive	0.42 kPa^−1^	70 ms	1–9 kPa	100 µm
[18]	Paper, Silver paste, CNTs	Resistive	2.56–5.67 kPa^−1^	30 ms	0–20 kPa	> 1 mm
[20]	Hemispheric microstructures, PDMS, Au, Ag Paste	Resistive	196 kPa^−1^	26 ms	0–100 kPa	-
[21]	Microstructures, PDMS, ITO‐PET, CNFs	Resistive	3.6 kPa^−1^	20–50 ms	0–2 kPa	> 1.5 mm
[22]	Pyramid microstructures, PDMS, PPy/PDMS, Au/Cr	Resistive	> 200 kPa^−1^	50 us	0.1–1,000 Pa	-
[23]	PDMS, BCZT particles	Piezoelectric	0.55 kPa^−1^	-	28.8 V output, 50–1,000 kPa	0.5 mm
[24]	Graphene field-effect transistor (GFET), Metal-insulator-metal (MIM)	Piezoelectric	0.00455 kPa^−1^	-	100 mV operation, 23.54–94.18 kPa	-
[25]	Silk fibroin, AgNWs, PDMS	Triboelectric	-	7 ms	~ 90 V output, 8–22 kPa	0.12 mm
[27]	PDMS, Paper, Graphite (Pencil)	Multi-Touch/Capacitive	0.62 kPa^−1^	> 200 ms	0.5–10 kPa	> 250 µm
[28]	Ni-coated textile, CNT, Acrylate polymers	Multi-Touch/Resistive	14.4 kPa^−1^	24 ms	0–15 kPa	-

### 2.1. Capacitive touch sensors

There are two main types of capacitive sensor technology [13,14], consisting of self-capacitive and mutual-capacitive sensors. A self-capacitive system measures changes in capacitance (with respect to earth ground). Considering a parallel-layer model, the electrode forms one layer of a capacitor, with the other layer being either ground or the user’s finger. A touch causes the electrode capacitance to increase, as the human body ‘adds’ capacitance to that of the system [15]. Mutual capacitance is the intentional or unintentional capacitance between two ‘charge holding objects’ [16]. Projected capacitance touch sensors intentionally create mutual capacitance between elements of columns and rows in the vicinity where each intersects the other. This structure allows the system electronics to measure each node (intersection) individually to detect multiple touches during one scan. Some touch devices use surface capacitance measurement to determine the human body (finger). A small voltage is applied to the sensor. When the finger comes into contact with the sensor, a capacitor is formed dynamically [2].

The capacitive element is mechanically simple and robust. So capacitive touch sensors are able to work over a wide temperature/pressure range. Because no direct current (DC) flows through the capacitor, they are inherently low power, small hysteresis, and suitable for wireless applications. However, the disadvantage of this sensor type is non-linearity and stray capacitance when having electronic devices close to the sensor [17].

### 2.2. Resistive touch sensors

Resistive touch sensors work as the pressure-sensitive sensors [18]. It made of several layers, the most important being two electrode layers separated by a low-conductive thin layer. Herein, the resistance changes predominantly as the result of the change in the contact resistance with lightly pressing. A resistive touch sensor can also sense the touch of objects other than the finger, such as a stylus. This is the earliest used type with linear output, fast response time, low cost, and high durability. However, the resistive-sensing type has to be powered; thereby, it is unsuitable for low power systems (wearable systems) [7].

One type of these sensors is based on piezoresistive materials, such as metals or semiconductors. These materials exhibit a change in electrical resistance (the distance between charged particles) when mechanical stress is applied [19]. Geometric microstructure designs can strongly increase the performance of sensors, such as pillars, hemispheres, and pyramids [14,20–22]. The pyramid microstructure is widely used because of its nonuniform stress distribution. For a given applied pressure, the tips of the pyramid shapes will compress more than other structures, resulting in higher mechanical deformations and sensitivities.

### 2.3. Piezoelectric and triboelectric touch sensors

A piezoelectric sensor is made by piezoelectric materials transforming deformation into electrical energy [23,24]. When a small mechanical pressure (lightly touch) is applied on certain so-called piezoelectric materials, the electric charges could be separated because of electrical dipole moments, and then an electrical voltage is generated. A triboelectric nanogenerator (TENG) utilizes triboelectrification (or contact-electrification) [25] to generate an electrical potential signal in response to physical contact (a touch) without reliance on an external power supply. Piezoelectric and triboelectric effects are often employed in *e*-skin or *e*-textile sensors. To date, there are many studies on circuit design and material aspects to enhancing the electric output, minimize the power loss caused by the signal irregularity, or optimize the contact surfaces. However, the practical applications are restricted due to low flexibility, stretchability, and poor dynamic performance. In these types, sensor elements are self-powered. So, the main advantages of piezoelectric touch sensors are robustness and low power. Meanwhile, the disadvantages are complex, and hard-to-integrate into a system.

Multi-touch is a technology that enables a surface to recognize the presence of more than one point of contact at the same time. Multi-touch functionality, mainly based on the sensor arrays, allows performing multiple finger gestures, such as swipe, scroll, select, zoom in, and zoom out. These sensor arrays can be fabricated by the capacitive, resistive, triboelectric, or optical principles. However, many studies demonstrated the capacitive or resistive sensor arrays are best suited for multi-touch surfaces [26–28].

## 3. Nanocomposite materials

Materials of flexible sensors should be lightweight, comfortable, biocompatible, and not cause irritation. Nanocomposite materials are most commonly, consisting of metallic thin films [29–31], metal nanowires (NWs) [32,33], carbon nanotubes (CNTs) [34–36], conductive polymers [37–39], and metal nanoparticles (NPs) [40–42]. Recently, the NWs, CNTs, and conductive polymers are preferentially used because of their abilities in the large active area, high electrical conductivity, and good electrochemical activity. Among that, NWs/CNTs composites can directly coat or print on substrates layers [40,43] to create highly sensitive, stretchable, and durable sensors. Besides, the conductive polymers can be synthesized by chemical or electrochemical deposition. The poly(3,4-ethylenedioxythiophene) (PEDOT), and especially its complex with poly(styrene sulfonate) (PEDOT:PSS) [39], show highly conductive, largely transmissive to light, processible in water, and highly ﬂexible.

Nearly all of the sensor parts can be fabricated by printing. Thus, the printable conductive materials are particularly advantageous for the mass-production. Among the investigated studies, the metal conductive inks show a promising prospect due to they can disperse in solvents to compatible with different printing technologies. Inks based on Ag nanoparticles and nanowires are most extensively studied as elastic electrodes/conductors for polyethylene (PE) [44,45]. Inks based on Cu nanoparticles, which are cheap and have a high conductivity, have also drawn much attention [46,47]. Besides, carbon nanomaterials-based inks (CNTs, graphene) show the printable and stretchable for flexible sensors [48–51].

Some touch display and photovoltaic applications require the use of highly transparent stretchable electrodes, which can be created by integrating indium tin oxide (ITO) [52], AgNWs [53,54], CNTs [55,56], graphene [57], and PDMS [55], PEDOT:PSS [58]. The major challenge of fabricating transparent electrodes/conductors is the tradeoff between conductivity or flexibility and transparency, which depending on conductive filler concentration [59]. This is especially true for touch sensors such as fingerprint sensors, touch screens.

Intrinsically flexible materials include ionic materials and liquid metals. Through electrochemistry technologies, ionic additives can improve the stretchability and conductivity of PEDOT:PSS (up to 4100 S cm^−1^ at 100% strain) [37], detect both positive and negative pressures from −60 to 20 kPa of soft sensors [60]. Liquid metals, specifically eutectic gallium–indium alloys (EGaln), have an intrinsic stretchability, high conductivity, and very small piezoresistivity. Thereby, they can be used to fabricate flexible circuits by the integration of room‐temperature liquid metals (RTLMs) and water‐soluble poly(vinyl alcohol) (PVA) [61], asymmetric force-sensors by hydrophilic polymer networks [62], soft sensors by 3D-printed rigid micro-bump [63]. The presence of liquid components will create reliability issues in dynamic applications. The embedding liquid metals into elastomers (PVA, hydrophilic polymer) will be a good solution.

## 4. Manufacturing technologies

The electromechanical properties and flexibility of touch sensors will be different due to the materials and manufacturing processes. Therein, the coating [64,65], printing [66,67], spinning [68,69], and transferring [70,71] are some method-keys of the current studies.

**Figure 2.**
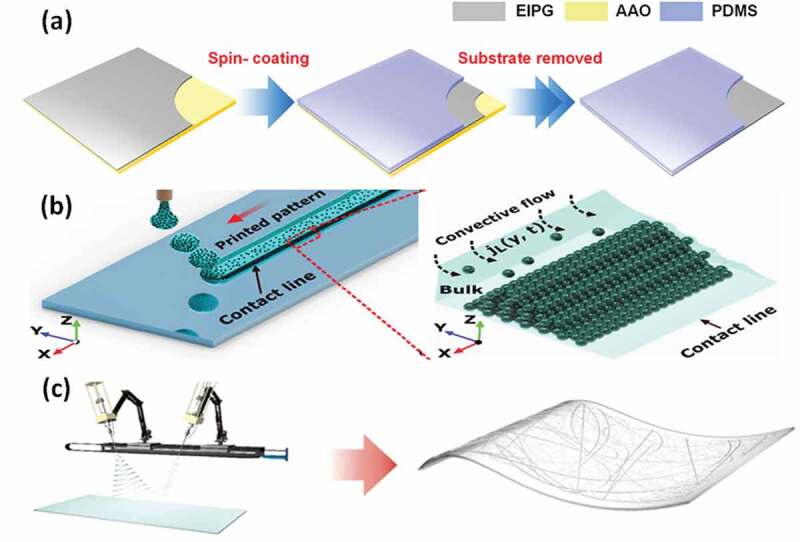
Schematic illustrations: (a) The flexible electrode using spin coating with electron-induced perpendicular graphene (EIPG), anodic aluminum oxide (AAO), and polydimethylsiloxane (PDMS). Reproduced with permission from Ref [4]. copyright 2020 Springer Nature. (b) The inkjet-printing controllable process for the evaporation-driven convective particle self-assembly at the contact line. Adapted with permission from Ref [73]. copyright 2020 Wiley-VCH. (c) The electrospinning and electrospraying processes of the AgNF-AgNW hybrid electrode. Adapted with permission from Ref [53]. copyright 2018 Springer Nature

Conductive coatings are applied to the yarns or surfaces. For example, through coating carbon nanofibers/graphene nanoplatelets (CnFs/GnPs) at both sides of a rubber piece, the stretchable capacitive touch sensors show a low sheet resistance value (≈ 10 Ω sq^−1^) and a thin nitrile rubber layer (60 µm) [72]. Besides, Chen et al. [4] created flexible touch sensors by electron‑induced perpendicular graphene sheets (20 x 20 mm^2^) embedded in porous carbon films (Figure 2a). These sensors have a fast response time (66 ms), high sensitivities (0.13 kPa^−1^ below 0.1 kPa, and 4.41 MPa^−1^ above 10 kPa). The silicon substrate is found to 0.5 mm.

Conductive printing (CP) refers to a type of flexible touch sensors that are fabricated by printing technologies. As shown in Figure 2b, the inkjet‐printing technique, creating ultra‐fine polydopamine (PDA) nanoparticle line arrays (NPLAs) with controllable line‐to‐line spacing (pitch size), is suggested to the transparent capacitive touch sensors [73]. The sensing area is 10 × 10 mm^2^ with a thickness of 380 µm (PET substrate). Meanwhile, an interesting application of the self‐powered touch sensor is able to light up some diodes, supply power to electronic devices, or charge capacitors. This paper-based energy harvester (~ 120 µm thick) is screen-printed using a mesh (carbons/silvers) [74]. Especially, 3D printing technology is attracting a great deal of attention in recent researches with a high commercialization ability as the large-scale and relative ease of integration. Yin et al. [75] proposed high-sensitivity wearable sensors from the 3D printing of ionic conductors. The materials are polyacrylamide/hydratable salts and polyethylene glycol (PEGDA). These sensors have a high sensitivity (0.84 kPa^−1^) at the pressure from 0.5 to 3 kPa. The structured films have a total thickness of 400 μm.

Electrospinning is the ultra-small fiber manufacturing method that uses electric fields, particularly suitable for making soft transparent metal electrodes. For example, a transparent and flexible fingerprint sensor array (Figure 2c) can detect tactile pressure and skin temperature [53]. In this approach, the thickness of the polyimide substrate is 25 µm. Herein, the multifunctional sensor array includes networks of hybrid nanostructures integrated ultra-long metal nanofibers and finer nanowires. Kweon et al. [76] presented a polymer-based pressure sensor. Therein, conductive core/shell polymer nanofibers consisted of poly(vinylidene fluoride-co-hexafluoropropylene) (PVDF-HFP)/poly(3,4-ethylene dioxythiophene) (PEDOT) (1 mm) are fabricated by three-dimensional (3D) electrospinning and vapor deposition polymerization methods. The working principle is based on the resistive sensor with high sensitivity (13.5 kPa^−1^).

Flexible sensor electrodes are also formed via transferring conductive materials onto elastomeric (textile or silicone). Basically, it is not a significant technological process. However, this approach is easy-to-use or becomes a support-step for other methods [71].

Besides, the controlled synthesis of materials as thin films, such as evaporation, is a fundamental step in many flexible devices. The evaporation often involves two basic processes: a hot source material evaporates and condenses on the substrate. For example, Vieira et al. [77] proposed a highly sensitive thermoelectric touch sensor based on p-type SnO_x_ thin film. Thermal oxidation films (the thickness of 60–160 nm, the deposition rate of 2 Å s^−1^, and the pressure of 2 × 10^−5^ mbar) deposited in borosilicate glass substrate was performed in the air-atmosphere at 250°C for 3 h. As a result, the touch sensor (from 60 nm-SnO_x_ thin films) achieves a high sensitivity ($V_{signal}/V_{noise}$ ≈ 20), with a rise time < 1 s.

## 5. Updates on touch-sensing

Considerable progress in flexible materials development for mechanically stretchable and bendable sensors will broaden the applications of ‘touch’s define’ in a new era. The morphological and mechanical properties of the conducting elements greatly affect not only their intrinsic electrical characteristics but also the performance and scope of applications of the touch types. Herein, we summarize researches and advances during the past two years in developing flexible sensors for *e*-skins, *e*-textiles, *e*-healthcare, and *e*-control approaches.

### 5.1. E-skins-based approach

Touch-sensing systems that mimic the stretchability and tactile sensing capability properties of human skin along with additional features are referred to as *e*-skins. Key application areas of the *e*-skins are in the skin attachable devices, prosthetics, and robotics. For small sustainable deformations, ultrathin materials-based *e*-skins such as poly(ethylene naphthalate) or poly(ethylene terephthalate) (PET) is a good choice. However, the elastomer substrates such as poly(polydimethylsiloxane) (PDMS), latex, and polyurethane (PU) are preferred for applications with stretchable requirements [78]. Asghar et al. [79] have demonstrated a piezo-capacitive flexible pressure sensor using magnetically grown microstructures (MPs/PDMS). The final size of each sensor was 250 mm × 250 mm × 1.5 mm. The device was capable of wide range pressure sensing (0–145 kPa) with ultrafast response time (50 ms), and high cyclic stability (> 9000 cycles).

**Figure 3.**
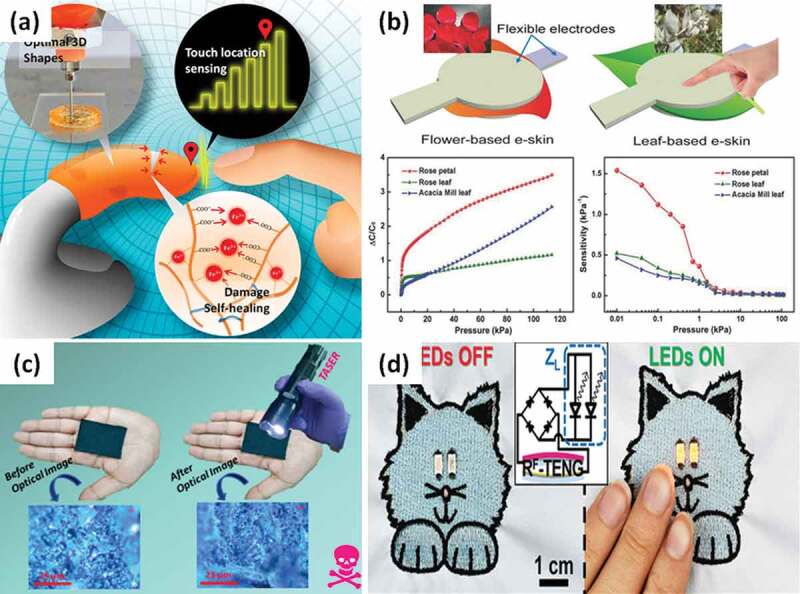
(a) Three-dimensional fingertip-shaped artificial skin device by 3D printing, which can sense exact touch location and heal mechanical damage spontaneously. Reproduced with permission from Ref [80]. copyright 2019 American Chemical Society. (b) Schematic illustration of the e‐skin consisting of two electrodes and the natural material (flower and leaf) as the dielectric layer in between. Besides, the graphs show the capacitance change and the sensitivity when applied pressure on three types of *e*-skins based critical point dried rose petal, rose leaf, and acacia leaf. Adapted with permission from Ref [81]. copyright 2018 Wiley-VCH. (c) Soft-touch textile sensors in high-voltage TASER test to short out the electrical shock. Adapted with permission from Ref [86]. copyright 2020 American Chemical Society. (d) Omniphobic triboelectric nanogenerators (RF‐TENGs) from *e*-textiles with the shape of a cat powering two LEDs embroidered as eyes (when touch). Adapted with permission from Ref [88]. copyright 2019 Wiley-VCH

Park et al. [80] developed a three-dimensional fingertip-shaped artificial skin device (Figure 3a), which has a large electronic signal contact upon touch by the capacitive sensing technology. It can sense the exact touch location and heal mechanical damage spontaneously. The touch device shows a good combination of 3D printing (nozzle size of 0.6 mm) and ion-conductive hydrogel. Besides synthetic materials, the natural plant materials, which simply consists of a dried flower petal or leaf (Figure 3b), can be directly used as the dielectric material in flexible capacitive *e*-skins [81]. The natural-material-based *e*-skins (thickness ~207 µm) operated in a broad-pressure range from 0.6 Pa to 115 kPa with a maximum sensitivity of 1.54 kPa^−1^, and high stability over 5,000 cyclic pressings, or bends. This is an interesting method for a green, cost-effective, and scalable approach.

During the operation, *e*-skins can wear and tear over time. One of the solutions to that is the use self-healing materials. Energy self-sufficiency can be created by incorporating phototransduction and photosensory functions referred to from the photosynthesis of plants [82]. The bioelectronic device is assembled by using a flexible top electrode comprising an indium tin oxide coated polyethylene terephthalate film (ITO-PET) and a gold‐coated PET film separated by a blend of photosynthetic protein and electron transfer mediator in a gap maintained by a double-sided adhesive spacer (250 μm). The ITO (200 nm) and gold films (20 nm) are deposited by magnetron sputtering. Herein, photosynthetic bioelectronic sensors on the *e*-skins show the ability to sense a touch stimulus (decrease to 3,000 Pa), low-intensity ultraviolet radiation (decrease to 0.01 mW cm^‐2^), and generate electrical energy (≈ 260 nW cm^‐2^). In another study, Zhao et al. [83] proposed flexible double-sided electronic skins based on the piezoresistive effect. The CNT sponge-based sensor (2 mm thick) achieved force direction detection by an ultrahigh sensitivity in a wide pressure range 0–4 kPa for 4015.8 kPa^−1^) and a rapid response time of 120 ms.

### 5.2. E-textiles-based approach

*E*-textiles are a new research direction and has great prospects in the future due to easy to approach and apply. Through adding electronic elements, *e*-textiles such as conductive fibers or fabrics can use in wearable devices, human-machine interface, or controlling/monitoring applications [84,85]. One kind of the dip-coated multifunctional textiles has been completed by uniform deposition of poly(3,4-ethylenedioxythiophene) (PEDOT) clusters and a stacked layer of PEDOTs/rGO-PEDOTs/PEDOTs within the wool/nylon textile surface [86]. As a result, the soft-touch textile sensors (Figure 3c) have high electrical conductivity (90.5 S cm-1), flexible electromagnetic interference shielding ability (73.8 dB), and high in-plane thermal conductivity (0.81 W/m·K) at minimum thickness (0.84 mm). Graphene is a popular material for *e*-textile sensors owing to its advantages over metal-based technologies. However, *e*-textiles of graphene oxide (rGO) have a high power consumption and poor electrical conductivity. To resolve these issues, Afroj et al. [87] applied a padding (pad−dry−cure) method in order to coat poly‐cotton textiles with graphene flakes. Those *e*-textile sensors provide a low sheet resistance (≈ 11.92 Ω sq^−1^) and could potentially create 150 m conductive textiles in just 1 min. The final thickness of graphene ‐coated (5 passes) and compressed poly‐cotton fabrics is found to 300 µm.

Some *e*-textiles are highly bendable, stretchable, and washable while keeping good electrical conductivity. As shown in Figure 3d, integrating the omniphobic triboelectric nanogenerators (RF‐TENGs) into the *e*-textiles shows excellent stability under deformations, washing durability, high sensitivity to touch, and cost‐effective manufacturing [88]. Thanks to natural and artificial fibers/fabrics such as cotton, silk, or polyacrylates, which are standard materials of life, *e*-textiles have a great advantage of comfortable for wearers. Alonso et al. [89] demonstrated graphene-enabled functional devices directly produced on textile fibers (0.03 mm thick and 2.4 mm wide). These capacitive touch sensors were fabricated by using a roll-to-roll-compatible patterning technique, opening new avenues for woven textile electronics.

### 5.3. E-healthcare-based approach

For healthcare applications, the devices need to have low energy consumption and good biocompatibility to avoid skin irritation. Some featured examples of the flexible touch sensing-based *e*-healthcare are wearable chemical and biochemical sensors. Their biggest challenge is the accuracy in a flexible working environment with many effects of humidity, temperature [90]. These chemical/bio-sensors are usually attached to human skin or into the garments/fabrics in order to7\7detect external dangerous agents or monitor the concentration of specific (blood) [91,92].

**Figure 4.**
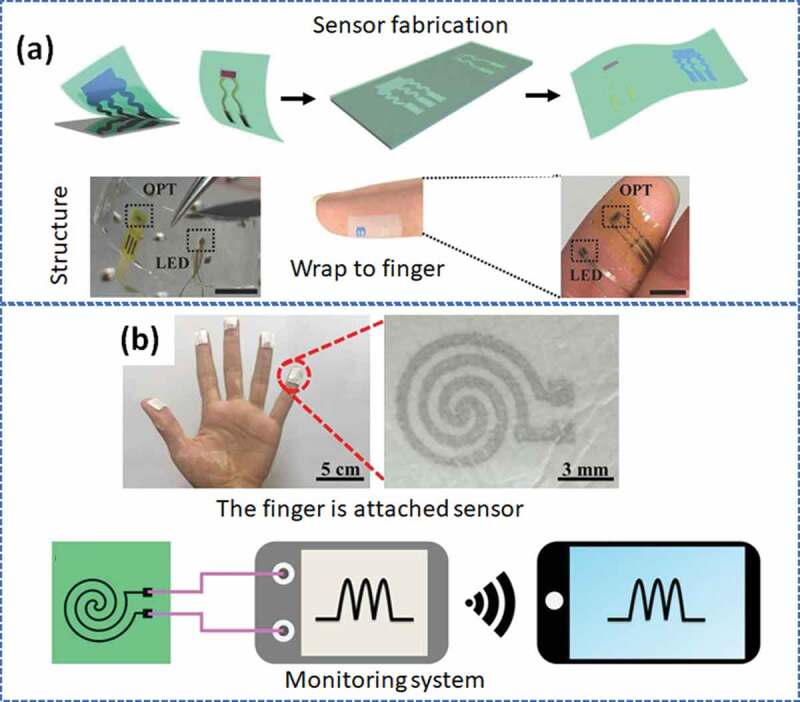
a) The photoplethysmogram (PPG) sensor hybrids an organic phototransistor (OPT) with an inorganic light‐emitting diode for cardiovascular monitoring, consisting of the fabrication process, the real picture of the sensor, and the OPT-LED structure are directly laminated onto the finger. Reproduced with permission from Ref [93]. copyright 2017 Wiley-VCH. b) The fingers of the hand are attached to the nanomesh film devices for tissue-temperature sensing and schematic illustration of remote monitoring of the sensing performance of the assembled film devices by wireless communication with a mobile phone. Adapted with permission from Ref [94]. copyright 2019 Wiley-VCH

Xu et al. [93] suggested a hybrid near‐infrared photoplethysmogram (NIR-PPG) sensor for cardiovascular monitoring. The structure of the NIR-PPG is a combination of a high‐sensitivity organic phototransistor (OPT) and a high‐efficiency inorganic light‐emitting diode. It is demonstrated that this sensor is capable of continuously monitoring heart rate variability (real-time) at low power (1.2 × 10^−15^ W Hz^−1/2^). Through the ultrathin encapsulation structure (1.8 µm), the device is highly flexible and allows transfer printing directly onto the finger (Figure 4a). In another example, a breathable electronic device can sense the tissue temperature at the wound site [94]. As shown in Figure 4b, the device is assembled from a crosslinked electrospun moxifloxacin hydrochloride‐loaded thermoresponsive polymer nanomesh film with a conductive pattern. The touch area has a size of 3 × 3.5 cm^2^, and a thickness of ~ 500 μm. Notably, this nanomesh film can also work as a highly efficient heater to trigger the release of antibiotics to eliminate bacterial colonization in the wound site when infected.

### 5.4. E-control-based approach

**Figure 5.**
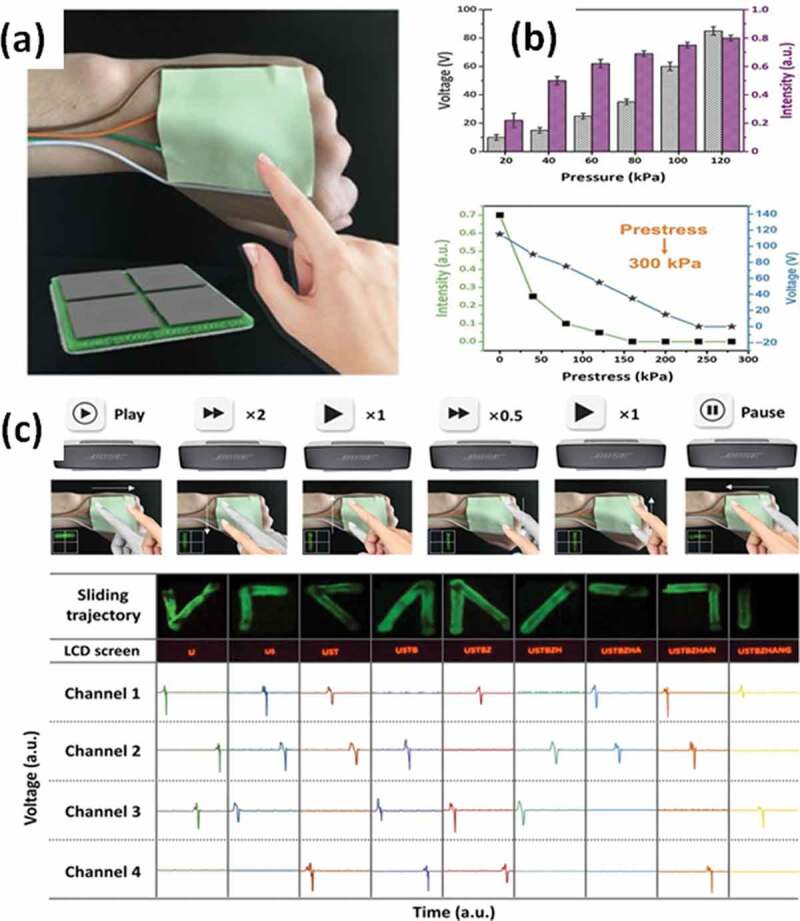
Application of the programmable touch operation platform based on the user-interactive *e*-skin (SUE-skin) for tracks recognition: (a) Optical photograph and four-electrodes schematic of the SUE-skin. (b) Light intensity and output voltage of the SUE-skin under different pressures and different prestress. (c) Touch operation demonstration for the audio controlling and the characters displaying. Adapted with permission from Ref [95]. copyright 2018 Wiley-VCH

Electronic controls by touch-sensing became popular devices today. Through soft structures and materials, those devices can able to bend or fold in order to easily integrate into different systems. A user-interactive electronic skin (SUE-skin) [95], which is based on a triboelectric-optical model, can convert the touch-stimulus into the electrical signals at low pressure (20 kPa), without an external power supply (Figure 5). By combining the SUE-skin with a microcontroller, this touch-platform recognizes more than 156 interaction logics for easy control of the consumer electronic devices. The SUE-skin can be folded, rolled, or bent without any mechanical failure, with a maximum folding force of 2 MPa and a minimum bending radius of 2 mm. In a bio-photocapacitive application for the visually impaired, Sai et al. [96] demonstrated a proof-of-concept six-pixel tactile sensor that created an auditory output from touch stimuli from braille codes. The primary structure of the device comprised six poly-dimethylsiloxane (PDMS) substrates (0.5 mm thick) wells to hold six ionic liquid droplets. This touch-to-audio braille reader contains photosynthetic pigment–proteins with a redox electrolyte in a liquid bridge to increase the sensory response. Besides, Kang et al. [97] reported a capacitive touch sensor with good sensing capabilities in both contact and non-contact modes, enabled by the use of graphene and thin device geometry. This 3D-touch sensor works on the principle of surface capacitive sensing with a thickness of 40 µm and a fast response time of 60 ms.

## 6. Summary and outlook

We have updated the latest researches on the flexible wearable sensors under view of touch-sensing. As above described, the development of new materials and technologies has expanded the capability, as well as the concept of touch sensors in many different applications. Important studies in the areas of *e*-skins, *e*-textiles, *e*-healthcare, and *e*-control are key-fields with enormous potential.

Besides inspiring advances, future works or efforts for commercialization still face a lot of challenges. We can list three main types of difficult issues, including material, technology, and working environment. For example, metal nanomaterials have relatively poor long-term stability, easy to oxidation, or desulfurization. Graphene and carbon nanotubes (CNTs) affect the immunological and induce inflammation of the lungs or interstitial fibrosis of asthma [98]. Metallic/metal oxide nanoparticles cause genotoxicity, a given type of multi-walled carbon nanotubes (MWCNTs) has been classified as a possible carcinogen [99]. Encapsulating the sensor’s area is a solution; however, it also will affect the performance [100]. Obviously, the encapsulation induces the thickness, thus decreases the flexibility. This also increases the necessary touch pressure to occur a change inside the sensors. The integration of flexible sensors with other flexible electronic devices or systems is not easy [101]. One problem is the power supply, which can support a continuously and fully-functional sensing system. There are some efforts to improve their flexibility and integration capability in the system [102,103]. Another challenge is the signal processing circuit. Most of the parts are embedded into an encapsulation (by silicone or PDMS). This approach can lead to unwanted noises or hot spots in contact with human skin [104]. Moreover, the working environment with the presence of noise, vapor, oils/sweat from the human body may cause false triggers. The technologies of collecting or analyzing data from touch-sensing should be optimized, such as distinguishable intended and false touches. The aforementioned challenges require multidisciplinary researches in finding solutions, aiming to design a complete touch-sensing system and bring products from laboratories to industrial production.
